# Humanism influencing the organization of the health care system and the ethics of medical relations in the society of Bosnia-Herzegovina

**DOI:** 10.1186/s13010-019-0082-7

**Published:** 2019-09-14

**Authors:** Ante Kvesić, Kristina Galić, Mladenka Vukojević

**Affiliations:** Mostar University Hospital, Bijeli Brijeg bb, 88 000 Mostar, Bosnia and Herzegovina

**Keywords:** Organization of medical care, Human ideals and values, Bosnia-Herzegovina

## Abstract

Every successful health care system should be based on some general humanistic ideals. However, the nationally organized health care systems of most European countries usually suffer from a deficiency in common ethical values based on universal human principles. When transitional societies, such as that of Bosnia-Herzegovina are concerned, health care organizational models are even more dysfunctional. The sources of a dysfunction in medical care system of Bosnia-Herzegovina are manifold and mutually controversial, including a lack of shared principles, an inappropriate involvement of politicians in medical care and practice, administrative difficulties arising from superficial communication systems, as well as economic limits concerned with the financing of health care. The deficiency of a moral culture of medicine, which is correlated to a general collapse of morality is also responsible for many problems affecting various aspects of life including medical care. Hence, medical ethics from a virtue perspective is becoming an important ingredient of any improvement deigned to provide better-quality medical care.

The aim of this paper is to underline the influence of humanism on the organization of health care systems and the ethics of medical interrelations in the society of Bosnia-Herzegovina. It is not intended to diagnose or resolve the problems, but to analyze them. It is also a critique of specific socio-political-economic influences on this health care system, inquiring if well-educated individuals in the virtues, which are involved in medical practice and education, would counteract them.

In conclusion, humanism creates a universal ethical structure, which is based on human values such as fidelity, trust, benevolence, intellectual honesty, courage, compassion and truthfulness. These values should represent the standard around which medical care is organized. Since the health care system in Bosnia-Herzegovina is not entirely founded upon humanistic ideals, addressing the socio-political-economic conditions that constantly undermine those values is a prerequisite for any much-needed improvements of the medical care.

## Introduction

Humanism is a philosophical and ethical standpoint, which emphasizes the value of human beings and privileges evidence and critical thinking. It is a progressive philosophy of life that affirms the ability and responsibility of a person to lead an ethical life of personal fulfilment, which aspires to the greater good [1]. Accordingly, we define humanism as a philosophy mainly based on rationalism and empiricism. Such a standpoint should influence the organization of the health care systems and the ethics of medical relations in every society, including Bosnia-Herzegovina to some extent in order to improve medical care for its inhabitants.

The system of health care in most European societies is organized at the national level, delegating the authority for its organization to the state itself [2]. Nevertheless, certain health care models occasionally suffer from various conditions, making them more or less dysfunctional in some measure, due to a lack of common ethical values based on universal human principles as first identified by Plato, Aristoteles and other great philosophers of Antiquity. When transitional societies, such as that of Bosnia-Herzegovina are concerned, organizational problems and pitfalls related to medical care are even more visible. Such societies are typically burdened with abundant economic and social problems, but also organizational and ethical ones, obscuring the quality of medical care, which is becoming increasingly side-lined.

Transitional societies are characterized by the redefinition of the societal classes, which were arbitrarily denied during the former communist systems. Health care systems in such societies are also affected by transition, although most countries have adopted some form of health insurance. Still, a high level of dependence on government subsidies exists to an overwhelming extent [3]. The same situation exists in the health care system of Bosnia-Herzegovina itself.

Every health care structure embodies a particular organizational culture, which affects people and reflects societal values, for good or ill. Such a structure is mainly constituted of intricate webs of relationships having many attributes relevant to ethics. However, it is regarded as a “health factory” in many societies including Bosnia-Herzegovina, where the number of beds, the number of patients processed, and the level of technical sophistication are more important in evaluations of the work done than the age, personal characteristics, religious beliefs and gender of patients. This bureaucratic approach remains omnipresent with little attention paid to ethical problems arising in the process of providing medical care. Therefore, the process of the institutionalization of bioethics is regarded as especially important to European societies in transition [4]. However, this process is constantly lacking in Bosnia-Herzegovina.

Recently, a considerable body of literature has explored medical ethics from a virtue perspective [5–8], while virtue has also received increasing attention in the field of medical education [9–13]. However, there is only a limited number of studies dealing with the concept of humanism in medicine.

The aim of this paper is to discuss the influence of humanism in the organization of health care systems and the ethics of medical relations in the society of Bosnia-Herzegovina. It is based on a literature review dealing with philosophical and medical ethical issues, as well as on our personal standpoints and experiences while working within the system of such a society. This paper is not intended to diagnose or resolve the problems, but merely to try to analyze them. It is primarily intended as a critique of specific socio-political-economic influences on the health care system of Bosnia-Herzegovina inquiring if well-educated individuals in the virtues, which are involved in medical care, practice and education, would counteract them.

## The conception of the good of the ancient philosophers and the physician-scientists and theologians of the middle ages

The famous ancient philosophers including Plato, Pythagoras or Aristotle along with first Christian theologians wrote about the conception of the good as the basic virtue to which other virtues were being upgraded. They defined virtue as a reference to the qualities of individuals to identify and pursue the good.

According to Plato (428/427 or 424/423–348/347 BCE), the most important idea of goodness is one that is identical to divinity. Accurately, the ‘Form of the Good’ sits atop Plato’s hierarchy of being and represents the ultimate foundation of his famou**s** metaphysics. His predecessor Pythagoras (c. 570-c. 495 BCE) advocated the abandonment of every evil and its replacement with good acts, identifying two souls, of which the *evil* one is derived from matter and the *good* one is derived from reason. Accordingly, the entire human life is the art of winning the soul for good over evil*.*

Aristotle (384–322 BCE) claimed that the characteristics of the good lie in every human being [14]. He also underlined that goodness equates to the excellence of a soul and not that of a body. For him, the virtues were the fundamental character qualities of an individual, and the inquiry into virtue was necessary to become good [15]. He firmly believed that virtues could be taught by example, expressing the teaching virtue as a moral practice.

Saint Augustine (354–430), who was an intellectual and a teacher with a wide spectrum of knowledge and skills of his time, wrote the most significant works in the Christian and theological literature. For him, the absence of the good was the counterbalance to evil. Saint Thomas Aquinas (1225–1274) stated that goodness is all that can be the subject of the tendency towards the good [16].

During the period of great mediaeval theologians, Islamic culture flourished outside Europe. Islamic scholars of that era made significant contributions in every aspect of art and science including medicine. Physician-scientists made substantial advances in medical care and built hospitals, where Jewish and Christian doctors, in addition to Muslim physicians, worked, allowing the poorest to benefit from the knowledge of outstanding doctors, regardless of their religion, age or gender. In these institutions, which provided the foundation for health care and medical education, distinguished authorities were engaged, including al-Razi (Rhazes) (865–925) from Baghdad. His writings provided the main medical curriculum for European schools into the fourteenth century. Simultaneously, Ibn Sina (Avicenna) (980–1037), an extraordinary Persian polymath, introduced an encyclopaedic approach to treatment in medicine, combining observations with medical information from Antiquity. These humanistic ideas provided the basis for medical care in Europe during its recovery from the Dark Ages [17].

Bearing all this in mind, one can wonder what happens with the intellectual, social and spiritual dimensions of the educated human being of the twenty-first century, who often lacks the feeling to be guilty of abuse, of neglect and/or of oppression. Obviously, there is neither awareness of the conscience and integrity of the individual, nor social awareness. As far as these two components are missing, spirituality certainly does not exist.

We defined a conception of the good as a vision of how various elements of a good life, such as friendship, material well-being, meaningful work or states of affairs, would ideally relate to one another and influence the society. We defined a value as a perception of moral wealth evaluated by an individual or an entire society and principles as general codes, which are helpful in creating fundamental truths. At the same time, we defined a virtue as universally recognized moral quality, which is an exhibition of someone’s good behaviour. Finally, we defined humanism as both a secular and a religious progressive philosophy based on reason, empiricism and ethical customs of community activities aligned with human needs and abilities.

## Philosophical and ethical background to European medical care

It is obvious that there is a strong philosophical and ethical background to European medical care originating from ancient philosophers, Arabic (Islamic) physician-scientists and Christian theologians, dating back to the Middle Ages, who advocated a morality, which was directly related to virtue. Such a morality is defined by what a virtuous person would do, and a virtuous person is one who does good acts [18]. The same can be said when medical professionals’ interpersonal relations are addressed, as well as when their relations with patients are concerned.

Personalism is an intellectual stance, as well as a philosophical and theological movement, which emphasizes the importance of the human person. It was introduced by Karol Józef Wojtyła, the future Pope Saint John Paul II (1920–2005), in 1969 [19]. According to strongly theological aspects of personalism, the dignity of the human person is derived from the fact that the human being is created by God. However, leaving aside the theological aspects of personalism, universal sociological aspects of such ideas are possible when ethics and human dignity are concerned. Therefore, when applied to various social environments including the medical environment itself, these ideas advocating the morality of a medical vocation, togetherness, solidarity and a respect for human dignity, become important for health*-*care professions. Thus, it could be a basis on which ethical foundations of modern and efficient health care systems are created. Still, it remains far from a ready-to-use approach for resolving imminent health-care problems.

The role of moral virtues among medical staff and in the physician-patient relationship is of the utmost importance too. Pellegrino describes medicine as the most scientific of the humanities and the most humane of the sciences [20]. Accordingly, positive human interpersonal relations in medicine and health care systems are precious for patients’ treatment outcomes, but also for the creation of an inspiring atmosphere between physicians and patients. Such relations are even more important, since the status of a medical working environment can influence the quality of medical care and the well-being of patients indirectly. The same can be said for the institutions responsible for providing medical education to a high academic level. Such an education of future medical professionals is vital for the creation of positive interpersonal relations at workplaces such as doctors’ practice offices and hospitals, as well as for originating the best in cooperation between doctor and patient based on high ethical standards. Consequently, it is proposed that the virtue of teaching should be an institutional mission of medical education [21]. Pellegrino and Thomasma described four levels of the good, which are important for the restoration of beneficence in health care: the biomedical good, the patient’s perception of the good, the good of the patient as a human being, and the spiritual good of the patient [6]. The dynamic of a healing relationship between the patient and physician is grounded on acting at all levels of the good [22]. Accordingly, fidelity, trust, benevolence, intellectual honesty, courage, compassion and truthfulness, as well as practical wisdom, are the professional virtues of a good physician, enabling medicine to become a true moral enterprise [23]. Therefore, medical personnel have to be virtuous in providing proper services to patients and securing their eventual healing as an ultimate goal of medical practice, knowledge and professional competence.

Taking the above into account, it is apparent that the origins of health care strategies have been established long before and were based on universal conception of the good. However, various above-mentioned philosophical positions are not necessarily interchangeable with each other, and the followers of these different perspectives are not expected to agree on every morality/ethics principle.

## Philosophical and ethical background to medical care in Bosnia-Herzegovina

Bosnia-Herzegovina is in the Balkans, located in South-eastern Europe, which has a small population and a unique culture, comprising different religions, beliefs and even academies. The Balkans’ negativity and personal interests often influence the spatial awareness of people in the country. In a broader explanation of the term, Balkan negativism would mean a political programme that is mostly backward, inevitable and inhumane. In the broadest sense, it would refer to everything that is negative and insufficient, affecting the economic, civilizational and scientific development of the countries in this particular geographic region. We believe that this universally acclaimed term has originated from the historical background of the region, which was dominated by different and conflicting cultural, religious, ethnic and political influences for centuries, having a predominantly negative effect on its societies’ development in turn.

We are a group of medical professionals and educators involved in the public health service of Bosnia-Herzegovina. This group is in daily contact with structural and ethical problems arising from the peculiarities of our health care system, which is burdened by grave obstacles originating from financial, organizational and poor interpersonal relations. Our experiences in medical working environment, where individuals in their access to work often harm other individuals and society in general on the personal and wider social level, inspired us to write this article.

Numerous personal instances in our own career can support the above-mentioned. We will try to explain some of them in order to illustrate the obstacles we are facing on an everyday basis. For example, one can meet all the necessary requirements and competences needed for scientific advancement in an academic career, but one can still be hindered in this pursuit by incompetent, politically powerful superiors. There may be no established ethical standards or judicial means of state institutions to protect such wrongdoing. The same can happen where someone’s job is concerned, since the employee may be put under enormous pressure by the political might of employers who are determined to restrict the pursuit of such a career for political and unprofessional reasons. Consequently, living in a time of rapid technological development and superficial communication, frequent efforts are made to simplify the interpersonal relations within the working environment. This often leads to the undermining of human values, such as knowledge, skills, abilities, talent, creativity and motivation.

It is impossible to observe humans’ life and work from an intellectual and physical level only. In a metaphysical sense, we live what we think. Therefore, what matters is what the individual thinks about working ethics and his/her colleagues in the form of threats, lies, discrimination and harassment. If we, as professionals and people responsible for education, allow this kind of social behaviour within our own environment to be acceptable, we favour the modelling of a negative identity for future generations of students, PhD candidates, professors or medical professionals. Accordingly, there is a question as to whether we form our reality on the dysfunctional foundations, beliefs and customs of our ancestors, or whether we leave even more damaging perspectives to our descendants? In fact, it is necessary to be aware that people with certain problematic working ethics try to devalue, annul and deny the majority of civilizational values achieved in the field of human rights for the sake of their own particular interests. Therefore, multi-layered, positively motivated and more moral people may leave a deeper impression in the creation of appropriate and stimulating labour relations and be competent to disagree with the one-sidedness of such relations.

Human interpersonal relations in the workplace are of the utmost importance to the outcome of the job being done, but also for the creation of a positive atmosphere among participants. When medical care is concerned, such relations are even more important, since the working environment can influence the quality of care and the well-being of patients indirectly.

The same can be said for the institutions responsible for providing medical education to a high academic level. Such an education of future medical professionals is vital for the creation of positive interpersonal relations in workplaces such as doctors’ practice offices and hospitals, as well as for originating the best in cooperation between doctor and patient, based on high ethical standards.

The potential of a young, educated man or woman cannot be developed to any adequate extent if there is a personal conflict in the workplace, characterized by the abuse of power and the imposition of force. However, people who fail to realize their own virtues cannot develop them alongside others. It is good that is useful for every individual in society. Virtue is knowledge: because of it, we should know how to do it right.

The maintenance and constant improvement of interpersonal relations in the workplace is an obligation and a duty of medical professionals and educators if the health service standards and the quality of medical care are to be advanced. It is important to underline that the high-value ethical standards of an individual represent the groundwork for establishing a successful professional career.

Health care systems are about people and relationships; when these are ignored, this can impose considerable strain on both providers and users of medical care, leading to unresolved issues and tensions as well as ethical problems [4].

In a specific health care system like that in Bosnia-Herzegovina, which is troubled by various political, economic and ethnic problems, the quality of such relations is even more exposed since professional and scientific progress has to be based on competence and positive abilities.

Therefore, it is our responsibility to promote goodness in interpersonal workplace relations, and to insist on fair career and knowledge promotion for every individual involved in providing health care to patients and academic learning to students. However, it is obvious that individuals’ morality, as well as professional and teaching virtues alone would not manage to eliminate the sources of dysfunction.

Nonetheless, we believe that the sources of a dysfunction in medical care system of Bosnia- Herzegovina are manifold and mutually controversial, including a lack of shared principles and an inappropriate involvement of politicians in medical care and practice. Additionally, abundant administrative difficulties arising from superficial communication systems, as well as economic limits concerned with the financing of health care are also very much to blame. Finally, the deficiency of a moral culture of medicine, which is correlated to a general collapse of morality, is a great burden to the society and is responsible for many imminent problems affecting various aspects of life including medicine and medical care itself.

## Conclusion

Humanism creates a universal ethical structure, which is based on virtues such as fidelity, trust, benevolence, intellectual honesty, courage, compassion and truthfulness. Such virtues should represent the standard on which the health care system is organized. The maintenance and constant improvement of interpersonal relations in medicine and beyond are an obligation and a duty of medical professionals and educators in the context of advancing the level of health service and the quality of medical care. In specific health care systems such as that in Bosnia-Herzegovina, which is troubled by various political, economic and ethnic problems, the quality of such relations is even more exposed.

We believe that any professional and scientific progress has to be based on competence and positive abilities. Therefore, it is our responsibility to promote goodness and virtue in interpersonal relations, and to insist on fair career and knowledge promotion for every individual involved in providing health care to patients and academic learning to students.

An awareness of human principles is a prerequisite for nurturing righteous, enduring personal and professional decisions affecting the outcome of patients’ treatment, as well as the prosperity of the health system and the whole of society.

The ethical aspects of medical relations are also exceptionally important in the process of overcoming the inherent shortcomings of interpersonal relationships between providers and consumers of health care, particularly in societies such as that of Bosnia-Herzegovina.

Universal humanistic values have to be implemented in all health care systems, including those in transitional societies, which are not always founded upon such values, making them less functional in turn. Therefore, recognizing as well as eliminating these aberrations is a requirement for any supposed improvement in medical care and the further development of the whole of society.

We do believe that health care systems in majority of transitional societies, including the one of Bosnia-Herzegovina are not entirely founded upon humanistic ideals or values, yet. If such values are reaffirmed in these societies more, than the system’s dysfunctions would be diminished, but not utterly eliminated. Therefore, addressing the socio-political-economic conditions that constantly undermine those values is a prerequisite for any much-needed improvements of medical care.

## Data Availability

Not applicable.

## References

[1] Copson A (2015). What is humanism? The Wiley Blackwell Handbook of Humanism.

[2] Bar-Yam Y (2006). Improving the effectiveness of health care and public health: a multiscale complex systems analysis. Am J Public Health.

[3] McKee M, Fister K (2004). Post-communist transition and health in Europe. BMJ.

[4] Borovecki A, Oresković S, Have H (2005). Ethics and the structures of health care in the European countries in transition: hospital ethics committees in Croatia. BMJ.

[5] Drane JF (1995). Becoming a good doctor. The place of virtues and character in medical ethics.

[6] Pellegrino ED, Thomasma DC (1993). The virtues in clinical practice.

[7] Oakley J, Cocking D (2001). Virtue ethics and professional roles.

[8] Toon P (2014). A flourishing practice?.

[9] Eckles R, Meslin E, Gaffney M, Helft P (2005). Medical ethics education: where are we? Where should we be going? A review. Acad Med.

[10] Coulehan J (2005). Today’s professionalism: engaging the mind but not the heart. Acad Med.

[11] Bryan C, Babelay A (2009). Building character: a model for reflective practice. Acad Med.

[12] Strachan K (2015). The virtues of medical ethics education. Res Medica.

[13] Kotzee B, Ignatowicz A, Thomas H (2017). Virtue in medical practice: an exploratory study. HEC Forum.

[14] Bielecki A, Nieszporska S (2017). The proposal of philosophical basis of the health care system. Med Health Care Philos.

[15] Olivieri HM (2018). *Recta Ratio Agibilium* in a medical context: the role of virtue in the physician-patient relationship. Philos Ethics Humanit Med.

[16] Augustine S (2017). Confessions.

[17] Edriss H, Rosales BN, Nugent C, Conrad C, Nugent K (2017). Islamic medicine in the middle ages. American J Med Sci.

[18] Pellegrino ED (2002). Professionalism, profession and the virtues of the good physician. Mt Sinai J Med.

[19] Wojtyła K (2017). Person and Act.

[20] Pellegrino ED, Engelhardt HT, Jotterand F, Pellegrino ED (2011). The most humane of the sciences, the most scientific of the humanities. The Philosophy of Medicine Reborn: A Pellegrino Reader.

[21] Shelton W (1999). Can virtue be taught?. Acad Med.

[22] Pellegrino ED (1988). For the Patient’s good: the restoration of beneficence in health care.

[23] Pellegrino ED (2012). Medical ethics in an era of bioethics: resetting the medical profession’s compass. Theor Med Bioeth.

